# Patient Preferences for Hospital Quality: Case Study of Iran

**DOI:** 10.5812/ircmj.12851

**Published:** 2013-09-01

**Authors:** Yasser Jouyani, Mina Bahrampour, Mohsen Barouni, Reza Dehnavieh

**Affiliations:** 1 Health Management and Economics Research Center, Facyulty of Health Management and Information Sciences, Tehran University of Medical Sciences, Tehran, IR Iran; 2 Medical Informatics Research Center, Institute for Futures Studies in Health, Kerman University of Medical Sciences, Kerman, IR Iran; 3 Research Center For Health Services Management, Institute for Futures Studies in Health, Kerman University of Medical Sciences, Kerman, IR Iran; 4 Research Center for Modeling in Health, Institute for Futures Studies in Health, Kerman University of Medical Sciences Kerman, IR Iran

**Keywords:** Patients’ Rights, Hospitals, Discrete Choice Experiment

## Abstract

**Background:**

Due to the importance and uniqueness of the characteristics of the health sector, one of the most important priorities of the Ministry of Health is measuring the efficiency and quality of services which are provided for the people who refer to the health sectors. In all health systems, responding to the needs and wishes of patients is a crucial priority.

**Objectives:**

The main purpose of this study is to prioritize the features of the services from the perspective of patients, by applying the Logit model.

**Materials and Methods:**

This study is a descriptive cross-sectional study and in terms of results it can be classified to an applied study. Data were collected by a questionnaire filled by 330 patients in Imam Khomeini hospital, and for estimating the utility function the software STATA version 10 was applied. In this study the preferences of patients who admitted to hospitals were identified by calculating the marginal utility of the characteristics, where we also used Marginal Rate substitutions (MRS).

**Results:**

Determination of the marginal utility characteristics shows that the first priority in receiving hospital services is the type of examination, and the last priority in the cleaning service of the sections and restrooms . Waiting time between hospital arrivals and admission has a negative sign which indicates a negative impact on patient preference.

**Conclusions:**

The results of this study are consistent with studies by Kara Hanson and Barbara Mc Clean, where in their study they also showed that by the patient’s perspective, hospital examination is the most important quality characteristic (coefficient = 2.78). In other words, the ultimate purpose of the hospital visit is the quality of service and examination, where many patients are willing to wait longer or pay higher costs to get the best services.

## 1. Introduction

In today's world, industrial and service organizations are competitive. Health centers and hospitals are also involved in this competition. An organization can be a winner in this competition if it recognizes the needs and demands of its customers by providing quality services that provides health and patient satisfaction (1).

Although health care is similar to other economic models in some ways, but overall a great difference can be seen between health care and other economic sections. Some of the characteristics that are well considered in the health care sectors can be characterized as irrational and senseless in other economic sectors. For example, uncertainty, asymmetric information, and adverse characteristics of individuals are just used in the health section. These features cause modeling the behavior of individuals in health centers to be different (2).

However, there are a lot of problems in the health system and hospitals, where the statistics and figures show that complaints on medical staff referred to Legal Medicine Organization of Tehran were 134 cases in year 1374,823 cases in year 1383, and reached 1270 cases in year 1384 (3).

Evaluating the efficiency and the quality of services provided for the public health section is the most important priority of the Ministry of Health, and studying patients’ satisfaction with hospital services quality is also an important method for evaluation of health services. The importance of patients’ satisfaction with health services quality will increase because the experience of illness and the need to comply with treatment and follow-up process will also increase as well as the need to provide more comprehensive support. Staff performance and their behavior help the patient feel more comfortable in the new situation. The staff behavior has a significant effect in reducing anxiety and encouraging the patients. In the medical services there are psychological needs on one hand and physical needs on the other hand. In this regard, due to the ultimate goals of the Islamic Republic of Iran to provide the best possible services for patients, assessing and prioritizing the needs of patients seems necessary. (4).

The world health report 2000 stated that consideration or respect to the patient, emergency and urgent care, reasonable wait times for non-emergency patients, or food and cleanliness are major duties of the health system. Hospitals, one of the main components of the national health systems, must try to identify the aspects of those duties and improve their performance. (5).

It is important to apply a discrete choice experiment (DCE) in prioritizing the characteristics according to patients’ perspective. DCE potentially is useful to extract the priorities in providing health care. This method estimates the importance of different features that health has and overall it measures satisfaction or benefit from the services. Discrete choice experiment can estimate the characteristics of the various services and how we evaluate these characteristics and their replacements (substitution).DCE depends on the following assumptions first, it assumes that each service can be described by different attributes and second they serve different characteristics depending on their nature.

DCE compared with direct measurement of willingness to pay have more important data acquired because it detects characteristics of products or services and shows how these characteristics affect the utility, and due to characteristics compared QALY has a better and wider framework for measuring the utility (6).

## 2. Objectives

Due to the uniqueness of characteristics of the health centers, and the importance of patient’s opinion and their association in the decision-making process, this research prioritizes the hospital services quality according to the patients’ point of view in terms of measuring the patients attitude and by also applying the logistic model. fo

## 3. Materials and Method

The main objective of this study is recognizing the patients’ priority to receive hospital services applying the Logit econometric model. The Logit(logistic regression)model is simply a non-linear transformation of the linear regression. The "logistic" distribution is an S-shaped distribution function which is similar to the standard-normal distribution (which results in a probit regression model) but easier to work with in most applications (the probabilities are easier to calculate). The logit distribution constrains the estimated probabilities to lie between 0 and 1.

Y=0.303+0.91x_1_+0.42x_2_+1.69x_3_+0.35x_4_+0.59x_5_

In the formula Y stands for utility and is our dependent variable; X is our independent variable; X_1_ stands for waiting time from arrival until hospital admission; X_2 _is for the handling of patients by medical staff at the hospital;X_3_ is for examination type;X_4 _is for personnel behavior; and X_5_ is for sector and toilets cleaning; and α is the Intercept.

This study is a descriptive cross-sectional study, and in term of the results it can be classified to an applied study, where the data were collected by a questionnaire. Designing of the questionnaire, characteristics, and different level of quality of hospital services was considered by reviewing previous studies, and the validity of it was confirmed by some of the experts. The questionnaire includes 10 scenarios related to each hypothesis. By comparing the two hypothetical hospitals, the two hospitals were different in some characteristics of the 5 characteristics; patients have to choose between the two hospitals. Given the patients tendency to choose between the hospitals we can prioritize the different characteristics of the patient’s point of view. After data collection, we used Stata version 10 to estimate the utility function. We calculated the marginal utility of the characteristics and preferences of patients who were admitted to hospitals in the research community and hospital services were identified as priority. When the final rate of substitution was calculated we could calculate the marginal rate of substitution (MRS).

Among the features, three cases had two different levels and two cases had three different levels that have caused a total of 72 scenarios. In order to convert the number of scenarios to an acceptable number in the questionnaire, the SPSS software, version 18 was used. By using the scenarios, hypothetical hospitals have been assumed, and by comparing them, the questionare was written. The questionnaire consisted of 10 comparisons between hypothetical hospitals. The sample size (N = 330) in this study, was estimated like previous studies by using the level of hospital utility.

Also a stratified model was used to conduct the sampling, so that all parts of the hospital related to the number of beds have been given an appropriate allocation and after that the samples were selected. Five features and their levels that were used in this study are shown in Table 1. 

**Table 1.** Characteristics of the hospital quality that are used in the study.

Feature name	Surfaces
**Waiting time from arrival until hospital admission**	Half an hour
	Two hours
	Five hours
**Handling of Patients by Medical Staff at the Hospital**	High
	Average
	Low
**Examination Type**	Full examination
	Incomplete examination
**Personnel Behavior**	friendly
	Indifferent
**Sector and Toilets Cleaning**	Frequently cleaning
	Sometimes cleaning

In the table we used Karen’s study for the waiting time from arrival until hospital admission hours, Kara’s study for the examination type and personnel behavior,and Tayary’s study for handling of patients by medical staff at the hospital and sector and toilets cleaning. 

## 4. Results

After collecting the data, it was found that men were more responsive individuals (57%) ,and (60%) percent of those respondents also have a minimum level of post-secondary education. In order to respond to the scenarios, patients had to completely understand the scenario. It was also clear that the majority of people selected hospital (A) for receiving services after comparing the scenarios. Patients’ care quality during treatment in hospital (B) is low, the staff behavior is not good, it does not have clean toilets nor a good sector cleaning service, but in hospital (B) the type of examination is a full kind and waiting time is minimum (half an hour).

After estimating the model by using Logit model, the estimation shows that among the study variables waiting time has a statistically significant relation in both levels, and also characteristics of the examination and waiting time are significant (P < 0.05),but two characteristics of patient's medical care and sector cleaning service are not statistically significant.

In qualitative research, for interpreting the relationship between each of the explanatory variables and dependent variables the term “ultimate effect” should be used conceptually. The coefficient used in these studies to assess the relationship between each of the explanatory variables and the dependent variable is not relevant. Conceptually, this effect shows the direction and rate of change in the explanatory variables caused by the changes in the dependent variable in each unit. This means that if a single change is made in any of the explanatory variables, it will show how much the dependent variable changes and in what direction will it be. Dependent variables are divided into two groups in some models; studying the computational R^2^ will not have a great value. Generally in these models computational R^2^ value is so much lower than 1 in time-series studies. The determination of coefficient is always high as well, because the same individuals are considered and the same trend was maintained over time for the same people,the correlation and determination of coefficient rises. Whereas in cross-sectional studies different individuals are considered at the same time, and by increasing the sample size the diversity of comments will increase and eventually the determination coefficient of model will reduce.

In cross-sectional studies the normal determination coefficients is 0.1 up to 0.3. So in this qualitative and cross-sectional study it is most likely to have a low determination coefficient. In this model, determination coefficient (R ^2 ^) is obtained as shown in Table 2. 

**Table 2.** Determination coefficients (R^2^)

Model	Coefficient
**Cragg-Uhler (Nagelkerke) R^2^**	0.188
**Efron's R** ^**2**^	0.142
**McFadden's R** ^**2**^	0.111
**McKelvey and Zavoina's R** ^**2**^	0.181
**ML (Cox-Snell) R** ^**2**^	0.140

To determine the significances, we apply the maximum likelihood estimation which has the chi-square distribution. The test shows that the likelihood ratio (LR) is significant.

LR determination and its formula are shown as the following:

LR=-2[L_unconditional_–L_conditional_]=339

The explanation is that the difference between the model with the only intercept (conditional model) and model with variables (unconditional model) is significant with 0.001 significances level, which means that these variables can be considered in the model.

**Table 3.** Logistic Regression

Number of obs	2250
**LR Chi Square (13)**	339.36
**Prob > Chi Square**	0.0000

To calculate the marginal effect of characteristics, we consider it as a fixed level (reference group) and measure the effect of changing from one level to higher levels, in utility (independent variable). The two characteristic that had three-levels (waiting time and the handling of patients by medical staff at the hospital) have different effects when shifting from first level to the second level or third level and of course the marginal effect of changing from the first to the third level is higher than the other one. In the following discussion the comparison of the marginal utility of the characteristics for three level variables shifting from the first level to the third level will be considered.

In the following table the marginal utility of the characteristics, which shows the patients preferences for visiting hospitals and receiving hospital services will be represented in order of priority.

**Table 4.** Variables of marginal utility.

Characteristics	Marginal utility
**Type of Examination**	0.3982713
**Waiting Time from Arrival until Hospital Admission**	0.214562-
**Handling of Patients by Medical Staff at the Hospital**	0.1038126
**Personnel Behavior**	0.087052
**Sector and Toilets Cleaning**	0.0144181

Calculation of the marginal utility of characteristics shows that the first priority in receiving hospital services is the type of examination, and sector and toilet cleaning has the least priority; the negative sign in waiting time from arrival till hospital admission shows the opposite effect of this character on patients preference, it means that with decreasing the waiting time patients’ utility for receiving hospital services will increase and vice versa.

## 5. Discussion

First of all the results of patients’ priorities for receiving hospital services will be presented by;

1) type of examination; 2) expected time of arrival until admission; 3) handling the patients by medical staff at the hospital; 4) personnel behavior; and 5) sector and toilets cleaning.In first view, we understand that the results of this research is consistent with the results of Kara Hanson and Barbara McClean study entitled “Preferences for hospital quality in Zambia” carried out using discrete choice experiment. Kara Hanson also came to the conclusion from the patients perspective that the quality of the examination is the most important quality characteristic (coefficient = 2.78) (7).

To explain the importance of examination we can consider that the main purpose of the patients’ visit to hospital is to receive good service such as good examination and patients’ treatment. In other words, we can say the main purpose of hospital visits are examination and receiving services, and other characteristics are the intermediate objectives of patients admitted to the hospital. So many patients are willing to wait longer or afford higher costs to get the best services. As in Axels et al. study entitled “Evaluation of patients’ preference for multiple myeloma therapy, a discrete choice experiment” showed that in this method patients are more interested in the qualitative aspects of services such as effective treatments, most lasting effects, longer life expectancy, and as well as fewer side effects.

Karen Gerard and her research colleagues show that the connection with the physician for a patient has 0.690 coefficient and has the first priority, having connection with the Nurse has 0.626 coefficient, and knowing the waiting time for admission has 0.005 coefficient and has the third place patients priority (8).

Ratcliff study (10) entitled “Assessing Patients preferences for characteristics associated with homeopathic and conventional treatment of asthma: A Conjoint -analysis study”, where the results displayedthat the patients’ priorities in this case are time to talk to the doctor who also listens to patients, andthe efficacy of travel expenses for treatment of asthma. These results are different from our study about “the patients' waiting time from arrival until admission to the hospital” that was ranked second among the patients, and it can be said that the waiting time is always an important issue, particularly for patients with acute illnesses. Although, in Kara Hansons’ , Tayarys’ (9), and Lancsers’ (11) study, waiting time did not have a high priority, but unlike these studies in our study waiting time has a high priority, perhaps it is due to the fact that our study was taken in a general hospital, with a large number of acute and emergency patients, and certainly compared to chronic patients, waiting times for emergency patients is more important. Increasing the waiting time from half an hour to five hours can largely reduce patients’ utility.
